# Molecular Evidences for the Interactions of Auxin, Gibberellin, and Cytokinin in Bent Peduncle Phenomenon in Rose (*Rosa sp.*)

**DOI:** 10.3390/ijms21041360

**Published:** 2020-02-18

**Authors:** Weikun Jing, Shuai Zhang, Youwei Fan, Yinglong Deng, Chengpeng Wang, Jingyun Lu, Xiaoming Sun, Nan Ma, Muhammad Owais Shahid, Yonghong Li, Xiaofeng Zhou

**Affiliations:** 1 Beijing Key Laboratory of Development and Quality Control of Ornamental Crops, Department of Ornamental Horticulture, China Agricultural University, Beijing 100193, China; jwk093@cau.edu.cn (W.J.); youwei@cau.edu.cn (Y.F.); SY20183172300@cau.edu.cn (Y.D.); cpwang@cau.edu.cn (C.W.); b20143010044@cau.edu.cn (J.L.); xmsun@cau.edu.cn (X.S.); ma_nan@cau.edu.cn (N.M.); owais@cau.edu.cn (M.O.S.); 2 School of Applied Chemistry and Biotechnology, Shenzhen Polytechnic, Shenzhen 518055, China; zhangshuai@szpt.edu.cn

**Keywords:** auxin, bent peduncle phenomenon, cytokinin, gibberellin, rose

## Abstract

In roses (*Rosa* sp.), peduncle morphology is an important ornamental feature. The common physiological abnormality known as the bent peduncle phenomenon (BPP) seriously decreases the quality of rose flowers and thus the commercial value. Because the molecular mechanisms underlying this condition are poorly understood, we analysed the transcriptional profiles and cellular structures of bent rose peduncles. Numerous differentially expressed genes involved in the auxin, cytokinin, and gibberellin signaling pathways were shown to be associated with bent peduncle. Paraffin sections showed that the cell number on the upper sides of bent peduncles was increased, while the cells on the lower sides were larger than those in normal peduncles. We also investigated the large, deformed sepals that usually accompany BPP and found increased expression level of some auxin-responsive genes and decreased expression level of genes that are involved in cytokinin and gibberellin synthesis in these sepals. Furthermore, removal of the deformed sepals partially relieved BPP. In summary, our findings suggest that auxin, cytokinin, and gibberellin all influence the development of BPP by regulating cell division and expansion. To effectively reduce BPP in roses, more efforts need to be devoted to the molecular regulation of gibberellins and cytokinins in addition to that of auxin.

## 1. Introduction

Peduncle morphology directly affects the yield and ornamental value of flowering plants. In roses, bent peduncle (BP) is a common phenomenon that substantially decreases the commercial productivity of plants. Interestingly, BP is always accompanied by a deformed sepal on the lower side of the peduncle, and the diameter of the peduncle is wider than normal [1]. The incidence of the bent peduncle phenomenon (BPP) varies with species and environmental conditions but is generally about 10–30% [1]. For example, the incidence of BPP in the cultivar ‘Beast’ in Korea can be as high as 20% in summer [2], whereas the annual BPP frequency in the cultivar ‘Peach Avalanche’ in China is in the 15–25% range.

The bending of plant organs results from asymmetric growth of the two sides of the organ and is known to be controlled by phytohormones [3], among which auxin (indole-3-acetic acid, IAA, and related compounds) plays a vital role. Auxin is synthesized mainly in the young shoot apex, leaf, and root tip and is then differentially distributed among tissues through polar transport [4]. Apical-hook development in *Arabidopsis thaliana* is divided into three stages: formation, maintenance and opening, then the formation of BP is accompanied by the formation of flower buds, and it will not open again. Dynamic and differential distribution of auxin controls cell division, cell expansion, and cell differentiation to modulate plant growth and development [4].

Auxin is perceived by co-receptors, the TRANSPORT INHIBITOR RESPONSE 1/AUXIN SIGNALING F-BOX PROTEINS (TIR1/AFBs) and Auxin/INDOLE ACETIC ACID (Aux/IAA), stimulating ubiquitination of Aux/IAAs by the E3 ligase complex and degradation by the proteasome complex, and subsequently regulating plant growth by activating auxin-response factors (ARFs) and promoting the transcription of auxin-responsive genes [4,5,6,7]. For example, DFL1 and YDK1, two auxin-responsive GH3 proteins in *Arabidopsis thaliana* (Arabidopsis), have been characterized by mutant analysis as negative regulatory factors affecting shoot and hypocotyl cell elongation and lateral root formation [8,9]. *ARF7* manipulates extensive cell growth by up-regulating the expression of *EXPA8* [10], thus leading to changes in cell wall properties [11,12].

Two other phytohormones, such as cytokinin (CTK) and gibberellin (GA), interact with auxin to regulate various growth patterns and developmental processes. Cytokinin signaling affects the auxin gradient in cambial cells, regulating meristematic activity [13]. Isopentenyl transferase (IPT), CTK nucleoside 5’-monophosphatephosphoribohydrolase (LOG), and members of the cytokinin oxidase/dehydrogenase protein (CKX) family are involved in CTK biosynthesis and degradation [14,15,16]. In Arabidopsis, *ckx3 ckx5* double mutants formed a stronger stem, with about a 15% larger diameter, compared with the wild type, corroborating the role of CTK in regulating stem size [17]. The CTK signaling-transduction pathway is a two-component system based phosphorelay that transmits a signal from the receptors to the downstream Arabidopsis response regulators (ARRs) through histidine. Among the ARRs, the expression of the type-A ARRs (ARR3–ARR9 and ARR15–ARR17) is rapidly induced by CTK, and the type-A ARRs, in turn, act as negative regulators of CTK signaling [18]. Type-A ARR proteins are reported to be regulated by a combinatorial mechanism involving both the CTK and proteasome pathways, thereby executing distinctive functions in plant growth and development [19].

The GA pathway acts as a key regulator of rose growth and development. Applications of GA1 and GA3 to axillary shoots in March inhibited floral development [20]. Previous study suggests that signals from the flower development stimulate GA biosynthesis in the sepals, which derive GAs appearing to be mainly involved in other development in rose [21,22]. The effect of GA on peduncle elongation of two rose cultivars (‘Nubia’ and ‘Mercedes’) was antagonized by combined application with cytokinin. Auxin and GA were involved in regulating peduncle strength (resistance to bending), which affected more strongly by auxin than by GA, and most strongly when auxin and gibberellin were combined [23]. Gibberellins [24] also modulate many developmental processes, including stem growth and hypocotyl elongation [25,26,27]. Genetic evidence suggests that GAs promote stem elongation in pea [28,29]. GAs are also reported to induce the expression of HOOKLESS 1 (HLS1) associated with ETHYLENE INSENSITIVE 3/EIN3-LIKE 1 (EIN3/EIL1), promoting apical hook formation in Arabidopsis [30]. The rice genes *OsGA3ox1, OsGA3ox2,* and *OsGA2ox3* encode proteins that are thought to catalyze the formation and accumulation of bioactive GAs [31,32]. A study in apple showed that MdGA20-ox is a key enzyme involved in GA biosynthesis and plays a significant role in vegetable growth, reducing the active GA content in RNAi-*MdGA20-ox* lines [33]. In tomato, GA2 oxidase 7, a class III GA 2-oxidase in the GA biosynthetic pathway, promotes internode elongation [34], suggesting a broader role of GA biosynthetic genes in organ-specific elongation.

In the present study, we investigated the transcriptional profile and cellular composition of BP in rose, uncovering the combined effects of auxin, cytokinin, and gibberellin on the BPP. These results should provide significant insight into the regulatory mechanisms underlying the development of bent organs in plants.

## 2. Results

### 2.1. Morphological Characteristics and Cellular Aspects of BP in Rose

**Figure 1:**
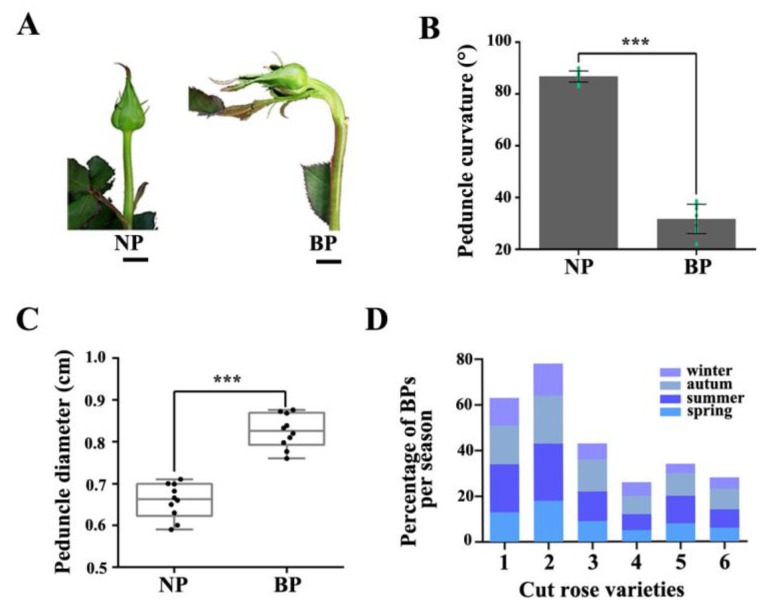
Characteristics of BPP. (**A**) Phenotypes of normal peduncle (NP) and bent peduncle (BP) at developmental stage 1. Scale bar = 10 mm. (**B**) Angles of NP and BP in the horizontal direction. (**C**) Statistics for the diameters of NP and BP. Error bars indicate standard deviation (SD). Ten biological replicates were performed for B and C (*n* = 10). Asterisks denote statistically significant difference determined by using Student’s *t*-test (*** *p* < 0.001). (**D**) Percentage of BPs per season of six rose cultivars. 1, ‘Peach Avalanche’; 2, ‘Awakening’; 3, ‘Jinhui’; 4, ‘Carola’; 5, ‘Chanel’; 6, ‘Ocean Song’.

**Figure 2:**
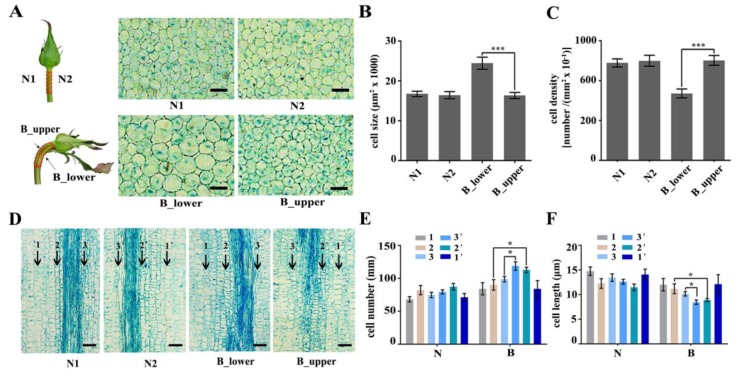
The cellular structure of BP. (**A**–**C**) Transverse sections of the two sides (**A**) and their respective cell size (**B**) and cell numbers (**C**) in NP and BP. (**D**–**F**) Longitudinal sections of the two sides (**D**) and their respective cell numbers (**E**) and cell lengths (**F**) in NP and BP. 1 and 1’ refer to parenchyma cells from the two sides, 2 and 2’ refer to primary phloem cells from the two sides, and 3 and 3’ refer to primary xylem cells from the two sides. Scale bars = 100 µm. Error bars represent SD. Three biological replicates were used. Asterisks denote statistically significant differences determined by using Student’s *t*-test (* *p* < 0.05, *** *p* < 0.001).

In the present study, we investigated six rose varieties (‘Peach Avalanche’, ‘Awakening’, ‘Jinhui’, ‘Carola’, ‘Chanel’, and ‘Song of Ocean’). The percentage of BPs in all varieties was the highest in summer and autumn. There were also significant differences in BP percentages among the six varieties, with Peach Avalanche and Awakening being the most prone to BPs, with incidence rates as high as 21% and 25%, respectively, in the summer months (Figure 1D). BP of rose is characterized by bending and increased diameter of the peduncle during development, accompanied by an enlarged sepal (Figure 1). To further investigate BP at the cellular level, we harvested the normal and bent rose peduncles, cut them along the central axis of the stem (resulting in samples N1 and N2 for the normal peduncles and B_upper and B_lower for the bent peduncles), and compared the morphology of their cortex parenchymal cells by paraffin section analysis (Supplementary Figure S1). Transverse sections of BP showed that the cells of B_lower were bigger than those of B_upper, whereas there was no obvious difference between cells from N1 and N2 (Figure 2A,B). Furthermore, the cell number of B_lower was significantly fewer than that of B_upper (Figure 2C). Subsequently, we compared the numbers of different types of cells in longitudinal sections of the same length between B_lower and B_upper (and between N1 and N2 as a control). There were significant differences between the BP (but not NP) samples from the two sides in the numbers of pro-phloem and pro-xylem cells, but not cortex parenchyma cells (Figure 2D–F). These results indicate that BP is caused by an asymmetry in the size and number of cells on either side of the central axis of the peduncle.

### 2.2. Transcriptome Sequencing of BP in Rose

**Figure 3:**
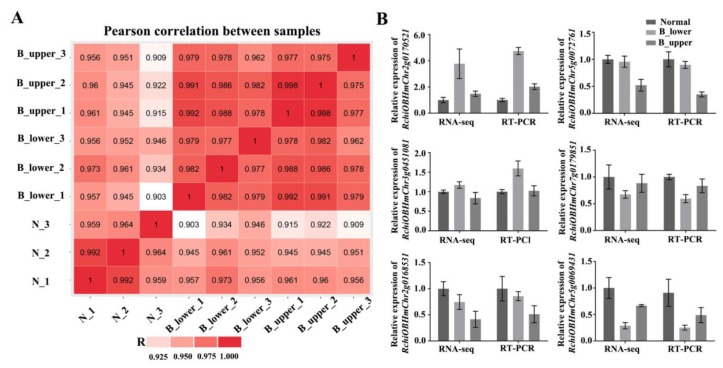
Validation of transcriptome data. (**A**) Pearson correlation between nine samples. (**B**) Relative expression of six selected genes (*RchiOBHmChr3g0451081*, auxin-response protein; *RchiOBHmChr5g0069431*, calcium/proton exchanger; *RchiOBHmChr2g0170521*, gibberellin-regulated protein; *RchiOBHmChr7g0179851*, ARR5-like; *RchiOBHmChr5g0072761*, expansion; *RchiOBHmChr2g0168531*, zinc finger) determined by qRT-PCR for validation of RNA-seq results. *RhUbi2* was used as an internal control. Error bars represent SD. Three biological replicates were performed.

To investigate the molecular mechanisms underlying BPP, we sequenced the transcriptomes of samples from NP and BP. We obtained the same two types of BP samples as before (B_upper and B_lower), as well as NP samples from along the central axis, at stage 1, in three biological replicates each, and used these to construct RNA-seq libraries (for a total of 9; Figure 2A). The RNA-seq analysis showed a total transcript length of 526,553,361 paired-end reads. De novo assembly of these high-quality cleaned reads generated 38,401 unique transcripts, with an average length of 2,021 bp (Table 1 and Supplementary Table S1). Calculating the Pearson correlation coefficients of the transcriptome profiles among the nine libraries and the biological replicates suggested the validity of our RNA sequence dataset with a high correlation (Figure 3A). To further validate the expression profiles of the RNA-seq data, we subjected six selected transcripts to further analysis by qRT-PCR. The results from the qRT-PCR analysis are generally consistent with the expression profiles obtained by RNA-seq, suggesting that the RNA-seq results were reliable (Figure 3B).

### 2.3. Dynamic Transcriptome of BP

**Figure 4:**
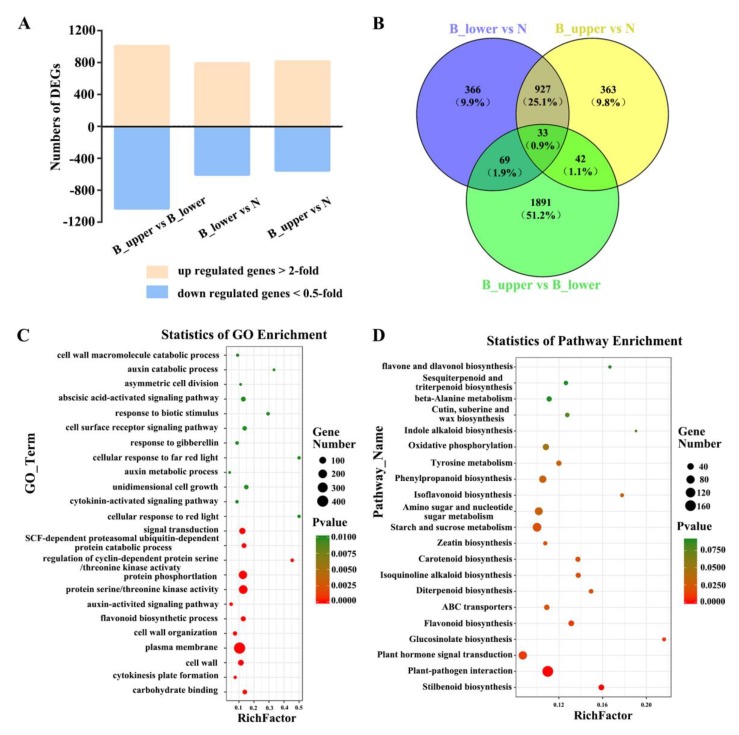
Numbers of DEGs between NP and BP and corresponding GO and KEGG enrichment data. (**A**) Numbers of up-regulated and down-regulated genes identified from comparison of B_upper and B_lower, B_upper and N, and B_lower and N. (**B**) Venn diagram of the data in A. (**C**) GO function enrichment analysis of DEGs. (**D**) KEGG pathway enrichment analysis of DEGs.

A total of 4,795 differentially expressed genes (DEGs) were identified between the two sides of the normal and bending peduncles at developmental stage 1 by comparing the transcript abundances of genes using a cutoff ratio of >2 or <0.5. The comparison of B_upper with B_lower yielded 1008 up-regulated and 1027 down-regulated DEGs; the comparison between B_upper and N yielded 813 up-regulated and 552 down-regulated DEGs; and the comparison of B_lower with N yielded 791 up-regulated and 604 down-regulated DEGs (Figure 4A). We identified a total of 2620 DEGs whose expression differed significantly between all three groups, and focused on these for further analysis (Figure 4B).

To evaluate the potential functions of these DEGs in the development of rose peduncles, we identified gene ontology (GO) terms for the DEGs. DEGs were classified into 24 GO function enrichment. The most significantly enriched GO terms included the following: ‘plasma membrane’, ‘protein serine/threonine kinase activity’, ‘protein phosphorylation’, ‘signal transduction’, ‘cell wall’, ‘auxin-activated signaling pathway’, ‘unidimensional cell growth’, ‘cell surface receptor signaling pathway’, ‘response to gibberellin’, and ‘cytokinin-activated signaling pathway’ (Figure 4C). DEGs were classified as enriched for genes involved in 21 KEGG pathways. These results revealed that genes involved in hormone pathways were the largest group, with 91 DEGs. In addition, the results of assigning DEGs to pathways in the KEGG database showed that ‘Plant-pathogen interaction’, ‘Starch and sucrose metabolism’, and ‘Plant hormone signal transduction’ were significant enriched among the DEGs identified (Figure 4D).

### 2.4. The Phytohormones Auxin, Cytokinin, and Gibberellin are Involved in BPP

Previous studies have established that auxin is involved in BPP [1,2], but the exact mechanism was unclear. Here, we determined that among the 96 hormone-related DEGs in the BP transcriptome, 36, 15, and 14 are involved in the auxin, cytokinin, and gibberellin pathways, respectively (Supplementary Figure S2, Table 2, Table 3 and Table 4). Therefore, we speculated that other factors besides these phytohormones may also affect BP development.

**Figure 5:**
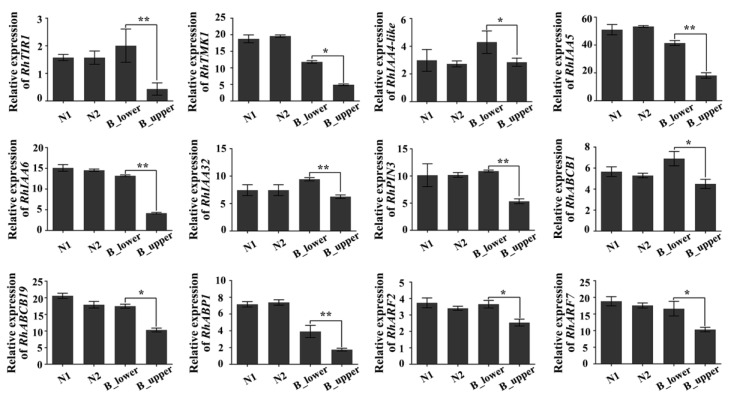
qRT-PCR of auxin-related genes in BP. Genes include those encoding auxin receptors (*RhTIR1*, *RhTMK1*), auxin-responsive factors (*RhIAA4-like*, *RhIAA5*, *RhIAA6*, *RhIAA32*), auxin transporters (*RhPIN3*, *RhABCB1*, *RhABCB19*, *RhABP1*) and auxin-regulated factors (*RhARF2*, *RhARF7*). Error bars represent SD. Three biological replicates were performed. Asterisks denote statistically significant differences determined by using Student’s *t*-test (* *p* < 0.05, ** *p* < 0.01).

To test this hypothesis this possibility, we first used real-time quantitative RT-PCR to assess the expression of genes involved in auxin signaling. The expression of RhTIR1, a key receptor in the auxin signaling pathway [35], was down-regulated in the upper side of BPs (B_upper) as compared to the lower side (B_lower), whereas there was no significant difference in *RhTIR1* expression levels between the two sides of NPs (N1 and N2) (Figure 5). Since a recent study of the auxin-TMK1 signaling pathway demonstrated that the receptor-like transmembrane kinase 1 (TMK1) could be triggered by high levels of auxin in Arabidopsis [36], we verified the expression of its homologue in rose. RhTMK1 expression was lower in the upper side than in the lower side of BPs (Figure 5). The expression of the other six auxin-response genes—*RhIAA4-like*, *RhIAA5*, *RhIAA6*, *RhIAA32*, *RhARF2*, and *RhARF7*—also showed the same trend (Figure 5). Furthermore, the expression of the auxin transport marker genes *RhABCB1*, *RhABCB19*, and *RhPIN3* was significantly reduced in the upper side of BPs (Figure 5). These results suggest that auxin signal transduction in the upper side of BPs is inactive compare to that in the lower side.

**Figure 6:**
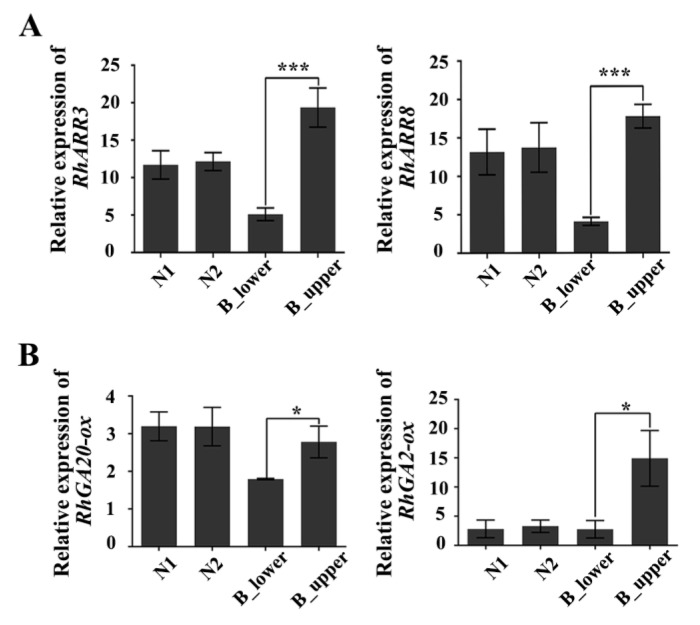
qRT-PCR of cytokinin- and gibberellin-related genes in BP. (**A**) Cytokinin-response genes *RhARR3* and *RhARR8*. (**B**) Gibberellin-synthesis-related genes *RhGA20-ox* and *RhGA2-ox*. Error bars represent SD. Three biological replicates were used. Asterisks denote statistically significant differences determined by using Student’s *t*-test (* *p* < 0.05, *** *p* < 0.001).

We also examined the expression of cytokinin- and GA-related genes in both sides of BPs. The expression of *RhARR3* and *RhARR8*, the rose homologues of Arabidopsis response regulator 3 (*ARR3*) and *ARR8*—two type-A response regulators rapidly induced by cytokinin [37]—was significantly greater in the upper vs. the lower side of BPs (Figure 6). Furthermore, *RhGA20-ox* and *RhGA2-ox*, two genes involved in GA biosynthesis, were also more highly expressed in the upper vs. the lower side of BP (Figure 6). These results indicate that there are significant different cytokinin and GA responses in the upper and lower sides of BPs.

### 2.5. The Effect of Enlarged, Deformed Sepals on BPP

**Figure 7:**
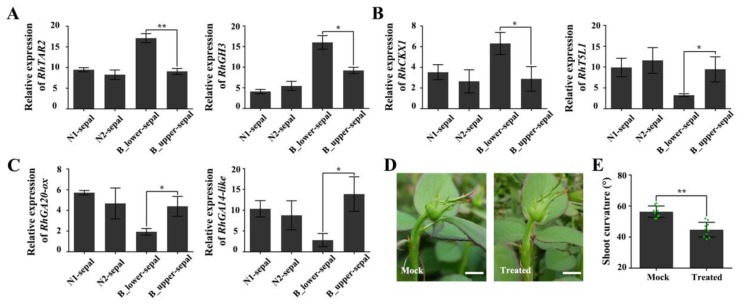
The expression of auxin-, cytokinin-, and gibberellin-related genes in deformed sepals determined by qRT-PCR. (**A**) Auxin-response genes *RhTAR2* and *RhGH3*. (**B**) Cytokinin-response genes *RhCKX1* and *RhT5L1*. (**C**) Gibberellin-related genes *RhGA20-ox* and *RhGA14-like*. (**D**) BP phenotype 3 days after removal of deformed sepals. Mock, plants with deformed sepals. Scale bars = 10 mm. (**E**) Statistics for the bending angle of peduncle 3 days after removal of deformed sepals. Error bars represent SD. Three biological replicates were performed for A, B, and C, and ten biological replicates for E. Asterisks denote statistically significant differences by using Student’s *t*-test (* *p* < 0.05, ** *p* < 0.01).

BP of rose is always accompanied by an enlarged, deformed sepal. To explore whether the deformed sepal is related to BP, we analyzed the expressions of genes in the auxin, cytokinin, and gibberellin signaling pathways in deformed (BPP) and normal sepals. The expression of the auxin-biosynthesis-related gene *RhTAR2* and the auxin-responsive gene *RhGH3* were both significantly up-regulated in the deformed sepals (Figure 7A). The transcript levels of *RhCKX1*, which encodes a protein that catalyzes cytokinin degradation, were also dramatically elevated in deformed sepals, whereas those of a positive regulator of cytokinin synthesis, *RhT5L1*, were lower in deformed sepals (Figure 7B). In addition, two genes involved in gibberellin synthesis, *RhGA20-ox* and *RhGA2-ox*, had decreased expression levels in deformed sepals (Figure 7C). These results suggest that signal transduction of auxin, cytokinin, and gibberellin in the deformed sepals is uncoordinated compared with that in normal sepals.

Next, we carried out an experiment in which we removed the deformed sepal from plants showing early signs of BP. This treatment somewhat reduced the degree of curvature at a later growth period (Figure 7D,E). We speculate that the hormonal response of the deformed sepals partially affects the response of the peduncle to hormones, resulting in unequal cell division and expansion on the two sides of the peduncle, and eventually in BP.

## 3. Discussion

### 3.1. BPP is a Physiological Disorder with High Frequency in Rose

Reports of the frequent occurrence of BPP affecting rose quality date back a decade, and BPP frequency has been shown to depend on varieties and seasons [1,2]. Previous studies had suggested that the BPP is caused by difference in auxin distribution in the two sides of the peduncle [1,2]. Our results showed that cytokinins and gibberellins are also involved in the development of BPs in addition to auxin (Figure 4, Figure 5 and Figure 6).

### 3.2. Auxins, Cytokinins, and Gibberellins Regulate the Cell Division and Expansion of BP

The structure of a BP is hook-like, mainly as a result of different growth rates of the two sides of the peduncle (Figure 1). Previous studies have established that relative differences in cell division and expansion on each side of the hook contribute to the bending [3]. Our results showed the cortex parenchyma cells on the lower side of a BP are much larger than those on the upper side, suggesting that they have undergone different rates of expansion (Figure 2A–C, and Supplementary Figure S1). Differential growth in higher plants, especially bending, is generally closely connected with auxin gradients in response to environment cues [38,39]. Therefore, we further investigated the involvement of auxin-related genes in bending growth by analyzing the expression patterns of auxin signaling pathway components in BPs. Both transcriptome analysis and qRT-PCR results indicate that the transcripts of these auxin-receptor, auxin transportation, and auxin response genes accumulate more at the lower side of BPs than those on the upper side (Figure 3, Figure 4 and Figure 5).

A recent report demonstrated TMK1 could specifically interact with and phosphorylate the repressors of the AUX/IAA family (IAA32 and IAA34) to maintain a stable rate under high levels of auxin, thereby regulating auxin-response genes and inhibiting growth [36]. It is consistent that protein phosphorylation is also enriched in the DEGs of RNA-seq. Accordingly, protein phosphorylation may be involved in the regulation of BP. Our results indicate that *RhTMK1* and *RhIAA32* expression were lower in the lower vs. upper side of BPs, and cell growth was clearly inhibited in the lower side. Further studies have shown that the polarity of PINs determines the direction of auxin flow [40]. In our study, we observed that the auxin signaling pathway factors, whether upstream receptors, downstream response factors, or transporter expression levels, were significantly less abundant on the upper vs. lower side of BPs (Figure 5). We speculated that different auxin responses on the two sides of a BP were the important cause of peduncle bending.

Longitudinal sections of paraffin sections indicate that pro-phloem and pro-xylem cells were more abundant in the upper vs. lower sides of BPs (Figure 2D–F). Cytokinins are well known as a key regulators of plant cell division [41,42]. In roses, two cytokinin-signaling genes, *RhARR3* and *RhARR8*, were up-regulated by cytokinin [37]. In this study, we identified 15 DEGs enriched in cytokinin signaling pathway by transcriptome analysis of BPs (Supplementary Figure S2, Table 4). Furthermore, we identified significant differences in the expression of RhARR3 and RhARR8 between the two sides of BPs (Figure 6A). We concluded that the cambial division activity on the upper side of BPs was higher than that on the lower side, possibly as a result of the differential distribution of cytokinin.

Gibberellins are known to control several aspects of plant growth and development, including stem elongation and leaf expansion [43]. The GA signal transduction pathway converts GA signal into gene expression and plant morphological changes. Mutant of the tomato GA biosynthetic related gene *GA2oxidase 7* has an elongated hypocotyl and internodes [34]. We suspected that differential expression of *RhGA20-ox* and *RhGA2-ox* on the upper and lower sides of BPs may be responsible for the different BP phenotypes in rose.

We identified some DEGs related cell wall pathways, including cell wall macromolecule catabolic process, cell wall loosening, cell wall organization, cell wall biogenesis and secondary cell wall biogenesis (Supplementary Table S2). Our results suggest that auxins, cytokinins, and gibberellins all likely participate in influencing cell-expansion proteins and cell-wall-related genes, thereby co-regulating cell expansion.

Due to the appearance of deformed sepals caused by external conditions, it continuously supplies endogenous IAA, which leads to the asymmetrical distribution of auxin on both sides of the peduncle, resulting in a high concentration of local auxin (in B_lower side), which has the effect of inhibiting growth.

In addition, the distribution of cytokinin and gibberellin on both sides is also different, the levels of cytokinin and gibberellin are higher in the B_upper side peduncle. These results indicated that the BPP is affected by interactions of phytohormones.

### 3.3. The Deformed Sepal Affects BP Development

The typically enlarged and deformed sepal of BPP, which appears as a leaf-like structure, may be identified as a case of phyllody [44], in which the largest sepal transforms into a leaf-like structure around the time when the apical bud becomes visible, positioned below the bud at a distance varying from about 0.5 to 3 cm (Figure 1A). It was previously speculated that deformed sepals might influence peduncle bending [2], although there was little evidence for this. Our results here indicate that the response to auxin, cytokinin, and gibberellin in the deformed sepals is very similar to that in the lower side of the BP, with higher expression of auxin-pathway genes and lower expression of cytokinin-and gibberellin-related genes compared to normal sepals. Moreover, the bending angle of the peduncle can be eased to a certain extent by removal of the deformed sepals (Figure 7D). Because endogenous auxin and gibberellin could be biosynthesized in young leaves [1,6,45], deformed sepals may be one of the hormone sources that affect the development of adjacent stems and vascular bundles.

In fact, we had detected the expression of marker genes used in peduncle in the sepal, petal and stem which was supplemented in Supplementary Figure S3. The results showed these marker genes have different expression pattern in peduncle, sepal, petal and stem. However, the expression of these genes had not significant difference between normal and abnormal sepal. At the same time, we found other different expression genes responded to phytohormones in deformed sepal (Figure 7A–C). We speculate that it is possible that the regulation of plant hormones in the deformed sepals is the first step of forming the BP, while the effect of plant hormones in the peduncle is the second step. So, the plant hormone regulation patterns in the peduncle and sepal are different.

## 4. Materials and Methods

### 4.1. Plant Material

Roses (*Rosa* sp., cv. Peach Avalanche) were grown in glasshouses in Tonghai County, Yuxi City, Yunnan Province, China. Normal and bending rose peduncles were harvested at developmental stage 1 (The diameter of bud was about 1 cm) and then divided into two parts along the central axis about 0.5 cm below the bud. The peduncles for RNA extraction were collected and immediately placed in liquid nitrogen and stored at −80°C. And the removed deformed sepal (B_lower-sepal), symmetrically normal sepals (B_upper-sepal), and N1, N2-sepals for RNA extraction and RT-PCR analysis were collected and immediately placed in liquid nitrogen and stored at −80°C. The peduncles for paraffin section were immediately placed in FAA solution. All samples for RNA-seq and RNA extraction were at the same developmental stage. There are three biological replicates.

The six cultivars are ‘Peach Avalanche’, ‘Awakening’, ‘Jinhui’, ‘Carola’, ‘Chanel’, and ‘Ocean Song’. All cultivars were standard cut roses and tetraploid. ‘Peach Avalanche’, bred by Lex Voorn (Netherlands, 2007); ‘Awakening’, bred by Jan Böhm (Czechoslovakia (former), 1935); ‘Jinhui’, a bud mutation cultivar of ‘Rouge’ (Yunxiu flower Co., Ltd., Yunnan, China, 2013); ‘Carola’, bred by Carmi Carmel (Israel, 1991); ‘Chanel’, bred by Takeo Kunieda (Japan, 1995); ‘Ocean Song’, bred by Rosen-Tantau (Germany, 2000).

### 4.2. Total RNA Extraction and RNA-Seq Library Preparation

RNA-seq was performed using samples of normal and bending peduncles (Figure 1). Total RNA was extracted using the hot borate method as previously described and treated with RNase-free DNase I (Promega) to remove any contaminating genomic DNA. Three biological repeats were performed for every part of the above peduncles. Strand-specific RNA libraries constructed by technology from the pooled RNA from the above samples were sequenced with the Illumina 4000 sequence platform. The raw sequence data have been submitted to the NCBI Short Read Archive with accession code PRJNA554000.

### 4.3. RNA-Seq Data Processing, Assembly, and Annotation

The raw data generated by sequencing were pre-processed by removing adaptor-containing sequences, poly-N, and low-quality reads; in addition, reads shorter than 150 bp were removed with *Q*-value ≤10. We aligned the resulting valid, clean reads to the rose genome (https://lipm-browsers.toulouse.inra.fr/pub/RchiOBHm-V2/) using the HISAT package, which allows multiple alignments per read (up to 20 by default) and a maximum of two mismatches when mapping the reads to the reference genome. The mapped reads were assembled using the StringTie software, and all the transcriptomes were reconstructed into a complete transcriptome by using Perl scripts. StringTie and edgeR were used to calculate expression levels of all transcripts. DEGs were analyzed by GO enrichment analysis (gene ontology) and KEGG signaling pathway enrichment analysis (Kyoto Encyclopedia of Genes and Genomes).

### 4.4. Paraffin Section

The peduncle materials were placed into FAA fixative solution (18: 1: 1 (v/v/v) 70% ethanol: glacial acetic acid: formalin), immediately vacuum-dried until the stem tissue was fully covered by the fixative, and then stored at 4 °C for at least 48 h. The experiment was repeated three times. The samples were then washed three times with distilled water, immersed in a 1:1 (v/v) mixture of 50% ethanol:70% glycerol, and rapidly vacuum-dried, and the experiment was repeated three times. Gradient dehydration, transparency, and ethanol embedding were then performed successively. Section (the thickness of section is 12 µm), dewaxing, dyeing (placed on glass and cut in 0.02% toluidine blue solution for 5 min), dewaxing again, sealing (Canadian gum, quickly sealing the residual toluidine blue with cover glass) and observation. Photoshop and ImageJ were used for cell counting and length measurements.

### 4.5. Quantitative RT-PCR

To validate the RNA-seq results, the transcript abundance of selected genes was analyzed by qRT-PCR using methods previous described [46]. Briefly, total RNA was isolated from samples with three biological repeats, and 2 µL of the first-strand cDNA was used as template with the Step One Plus TM real-time PCR system (Applied Biosystems) using KAPA™ SYBRR FAST quantitative PCR kits (Kapa Biosystems). The *RhUbi2* housekeeping gene was used as standard, which was validated in previous studies [47,48]. The primers used for determining transcript abundance are listed in Supplementary Table S3.

### 4.6. Removal of Deformed Sepal

We removed the deformed sepal of BP slightly with a blade and without damaging the stem. Hold the peduncle parallel to the leaf with your hand and remove the deformed sepal of BP slightly with a blade when diameter of flower bud is about 1 cm (developmental stage 1). Remove the deformed sepal when diameter of flower bud is about 1 cm (developmental stage 1). At the same time, the removed deformed sepal (B_lower-sepal) and symmetrically normal sepals (B_upper-sepal) for RNA extraction and RT-PCR analysis were collected immediately.

## 5. Conclusions

The results of our study reveal that phytohormones auxin, cytokinin, and gibberellin all play a role in regulating the BPP of rose.

## Figures and Tables

**Table 1:** Summary of rose peduncles parts transcriptome sequencing database.

Items	Total
No. of reads	398,886,448
No. of valid reads	395,462,558
No. of mapped reads	334,973,525
No. of assembled transcripts	260,541
Average length of transcripts	2021 bp
Total length of transcripts	526,553,361 bp

**Table 2:** DEGs involved in the auxin pathway.

Gene ID	FC	Log_2_(FC)	Up/Down Regulation	Annotation
**Response to auxin**				
RchiOBHmChr1g0342931	3.94	1.98	up	Carotenoid cleavage dioxygenase 7
RchiOBHmChr3g0478151	4.25	2.09	up	Ubiquitin-like protein Nedd8
RchiOBHmChr3g0478171	2.96	1.57	up	Ubiquitin-like protein Nedd8
RchiOBHmChr4g0392981	2.03	1.02	up	Transcription factor MYB15
RchiOBHmChr4g0424331	2.23	1.16	up	Transcription factor MYB12
RchiOBHmChr5g0056751	3.13	1.65	up	Auxin-induced protein 15A-like
RchiOBHmChr6g0268541	26.95	4.75	up	Auxin-responsive protein SAUR50-like
RchiOBHmChr6g0305471	10.96	3.45	up	Auxin-responsive protein SAUR71-like
RchiOBHmChr7g0210641	2.20	1.14	up	Auxin-responsive protein SAUR14
RchiOBHmChr7g0210711	40.28	5.33	up	Auxin-responsive protein SAUR3
RchiOBHmChr7g0210731	40.28	5.33	up	Auxin-responsive protein SAUR20
RchiOBHmChr7g0210801	1.40	0.48	up	Auxin-responsive protein SAUR23
RchiOBHmChr1g0324921	0.21	−2.25	down	Auxin-induced protein X15
RchiOBHmChr1g0362991	0.30	−1.75	down	Indole-3-acetic acid-induced protein ARG7-like
RchiOBHmChr4g0422041	0.36	−1.49	down	Flowering-promoting factor 1-like protein 3
RchiOBHmChr5g0048841	0.36	−1.47	down	Vacuolar-related NAC-domain 6
RchiOBHmChr7g0202161	0.40	−1.32	down	Auxin-responsive protein SAUR36-like
RchiOBHmChr7g0209651	0.21	−2.27	down	Auxin-responsive protein SAUR40
RchiOBHmChr7g0188911	0.60	−0.75	down	Auxin-responsive factor 2 ARF2
RchiOBHmChr2g0095551	0.72	−0.48	down	Auxin-responsive factor 7 ARF7
RchiOBHmChr6g0254401	0.67	−0.58	down	Auxin-responsive GH3 family protein GH3
**Auxin-activated signaling pathway**				
RchiOBHmChr4g0428791	2.35	1.23	up	Auxin-responsive protein 31 IAA31
RchiOBHmChr6g0311541	2.37	1.25	up	BIG GRAIN 1-like B
RchiOBHmChr7g0224691	2.24	1.16	up	Arabidopsis SKP1 homologue 1 ASK1
RchiOBHmChr3g0451081	0.49	−1.03	down	Indole-3-acetic acid inducible 4 like IAA4-like
RchiOBHmChr4g0389611	0.57	−0.80	down	Indole-3-acetic acid inducible 5 IAA5
RchiOBHmChr2g0137311	0.52	−0.94	down	Indole-3-acetic acid inducible 6 IAA6
RchiOBHmChr4g0434391	0.55	−0.87	down	Indole-3-acetic acid inducible 32 IAA32
**Auxin homeostasis**				
RchiOBHmChr2g0132571	2.92	1.55	up	Shikimate O-hydroxycinnamoyl transferase-like
RchiOBHmChr2g0132651	3.35	1.74	up	Shikimate O-hydroxycinnamoyl transferase-like
RchiOBHmChr5g0066541	0.35	−1.52	down	Shikimate O-hydroxycinnamoyltransferase-like
RchiOBHmChr6g0302041	0.20	−2.30	down	Transcription factor SHI RELATED SEQUENCE 1-like
**Auxin biosynthetic process**				
RchiOBHmChr6g0266901	0.45	−1.16	down	Transcription factor SHI RELATED SEQUENCE 3-like
RchiOBHmChr7g0192941	0.25	−2.01	down	Trytophan aminotransferase related 2 TAR2
RchiOBHmChr6g0292661	0.56	−0.84	down	Transport inhibitor response 1 TIR1
RchiOBHmChr3g0492431	0.57	−0.80	down	Receptor-like transmembrane kinase I TMK1
**Cellular response to auxin stimulus**				
RchiOBHmChr1g0318281	0.32	−1.62	down	Transcription factor agamous-like MADS-box AGL15
RchiOBHmChr4g0389411	0.01	−7.00	down	Enhancer of mRNA-decapping 4-like
**Auxin catabolic process**				
RchiOBHmChr2g0096111	10.10	3.34	up	2-Oxoglutarate-dependent dioxygenase DAOlike
**Auxin metabolic process**				
RchiOBHmChr4g0444701	2.33	1.22	up	IAA-amino acid hydrolase ILR1-like 4
**Auxin transport**				
RchiOBHmChr6g0305141	0.37	−1.45	down	UDP-glucose 4,6-dehydratase
RchiOBHmChr4g0441311	0.63	−0.67	down	PIN-FORMED 3 PIN3
RchiOBHmChr3g0470311	0.70	−0.52	down	ATP-binding cassette B1 ABCB1
RchiOBHmChr2g0168761	0.59	−0.77	down	ATP-binding cassette B19 ABCB19
RchiOBHmChr6g0291961	0.68	−0.57	down	Auxin binding protein 1 ABP1
RchiOBHmChr2g0162401	2.26	1.17	up	Serine/threonine kinase

FC, fold change.

**Table 3:** DEGs involved in the gibberellin signaling pathway.

Gene ID	FC	Log_2_(FC)	Up/Down Regulation	Annotation
**Gibberellic acid-mediated signaling pathway**				
RchiOBHmChr2g0159111	2.88	1.53	up	Transcription factor MYB101
RchiOBHmChr6g0309121	36.45	5.19	up	Gibberellic acid stimulated Arabidopsis 10
RchiOBHmChr2g0156431	0.44	−1.20	down	F-box protein SNE
RchiOBHmChr2g0170521	0.34	−1.57	down	Gibberellin-regulated 14-like GA14-like
**Response to gibberellin**				
RchiOBHmChr1g0378621	2.27	1.18	up	Transcription factor WRKY 27
RchiOBHmChr4g0444681	5.42	2.44	up	Gibberellin-regulated 3-like
RchiOBHmChr5g0062851	2.18	1.12	up	Transcription factor MYB48-like
**Gibberellin biosynthetic process**				
RchiOBHmChr1g0353791	3.41	1.77	up	Gibberellin 20 oxidase GA20-ox
RchiOBHmChr2g0089721	0.17	–2.58	down	Gibberellin 3-beta-dioxygenase 1-like
**Gibberellin catabolic process**				
RchiOBHmChr3g0486971	2.30	1.20	up	Gibberellin 2-beta-dioxygenase GA2-ox
RchiOBHmChr1g0318281	0.32	–1.62	down	Agamous-like MADS-box AGL15
**Cellular response to gibberellin stimulus**				
RchiOBHmChr7g0202321	2.07	1.05	up	Acid beta-fructofuranosidase 2, vacuolar
**Regulation of gibberellic acid-mediated signaling pathway**				
RchiOBHmChr6g0302041	0.20	–2.30	down	SHI RELATED SEQUENCE 1-like
**Gibberellin metabolic process**				
RchiOBHmChr5g0003991	3.35	1.75	up	Gibberellin 2-beta-dioxygenase 8 GA2-ox8

FC, fold change.

**Table 4:** DEGs involved in the cytokinin signaling pathway.

Gene ID	FC	Log_2_(FC)	Up/down regulation	Annotation
**Response to cytokinin**				
RchiOBHmChr7g0238791	12.27	3.62	up	Inosine-5 -monophosphate dehydrogenase 2-like
RchiOBHmChr4g0415601	0.16	−2.63	down	BAHD acyltransferase At5g47980-like
RchiOBHmChr4g0415621	0.16	−2.66	down	BAHD acyltransferase At5g47980-like
RchiOBHmChr4g0431311	0.24	−2.08	down	Deacetylvindoline O-acetyltransferase
RchiOBHmChr5g0048841	0.36	−1.47	down	NAC transcription factor 29-like
RchiOBHmChr6g0284071	0.41	−1.27	down	Wound-induced basic protein
RchiOBHmChr6g0304241	0.36	−1.47	down	Vinorine synthase-like
RchiOBHmChr4g0444831	1.79	0.84	up	A-type response regulator 3 ARR3
RchiOBHmChr2g0125351	1.56	0.64	up	A-type response regulator 8 ARR8
RchiOBHmChr1g0370561	1.67	0.74	up	TMO5-LIKE1 T5L1
**Cytokinin-activated signaling pathway**				
RchiOBHmChr1g0358681	2.82	1.5	up	Ethylene-responsive transcription factor CRF4-like
RchiOBHmChr5g0061201	0.19	−2.40	down	Histidine-containing phosphotransfer 1-like
RchiOBHmChr6g0250591	0.37	−1.42	down	Histidine kinase CKI1-like
RchiOBHmChr6g0250601	0.27	−1.89	down	LYSM-containing receptor-like kinase 1
**Positive regulation of cytokinesis**				
RchiOBHmChr4g0410231	2.26	1.17	up	NEDD1 protein
**Cytokinin catabolic process**				
RchiOBHmChr1g0319331	0.61	−0.72	down	Cytokinin oxidase/dehydrogenase 1 CKX1
**Cytokinesis by cell plate formation**				
RchiOBHmChr1g0341961	2.11	1.08	up	Tubulin binding cofactor A
RchiOBHmChr1g0361251	3.10	1.63	up	HAUS augmin-like complex subunit 2
RchiOBHmChr3g0495401	2.02	1.02	up	Cytoskeleton-associated protein 5

FC, fold change.

## Supplementary Materials

The following are available online at https://www.mdpi.com/1422-0067/21/4/1360/s1.
