# Supplementary material for: Molecular Evidences for the Interactions of Auxin, Gibberellin, and Cytokinin in Bent Peduncle Phenomenon in Rose (Rosa sp.)
Source: Int J Mol Sci. 2020 Feb 18;21(4):1360. doi: 10.3390/ijms21041360 (PMC7072929; doi:10.3390/ijms21041360)
Supplement: Supplementary file 1 [file ijms-21-01360-s001.zip › ijms-684956-supplementary.docx]

**Supplementary Materials**


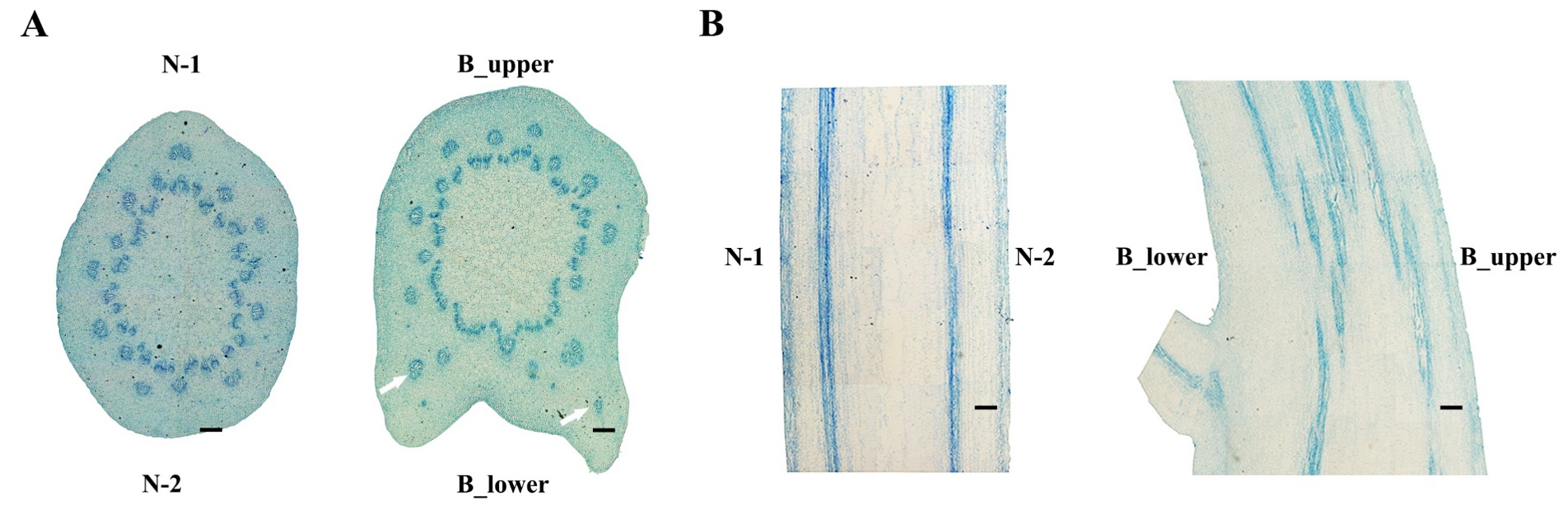


**Supplementary Figure 1. Transverse and longitudinal paraffin sections of normal peduncle (NP) and bent peduncle (BP).** A, transverse paraffin sections of NP and BP. The white arrows indicate additional vascular bundles in BP. B, longitudinal paraffin sections of NP and BP. Scale bar=200 μm.


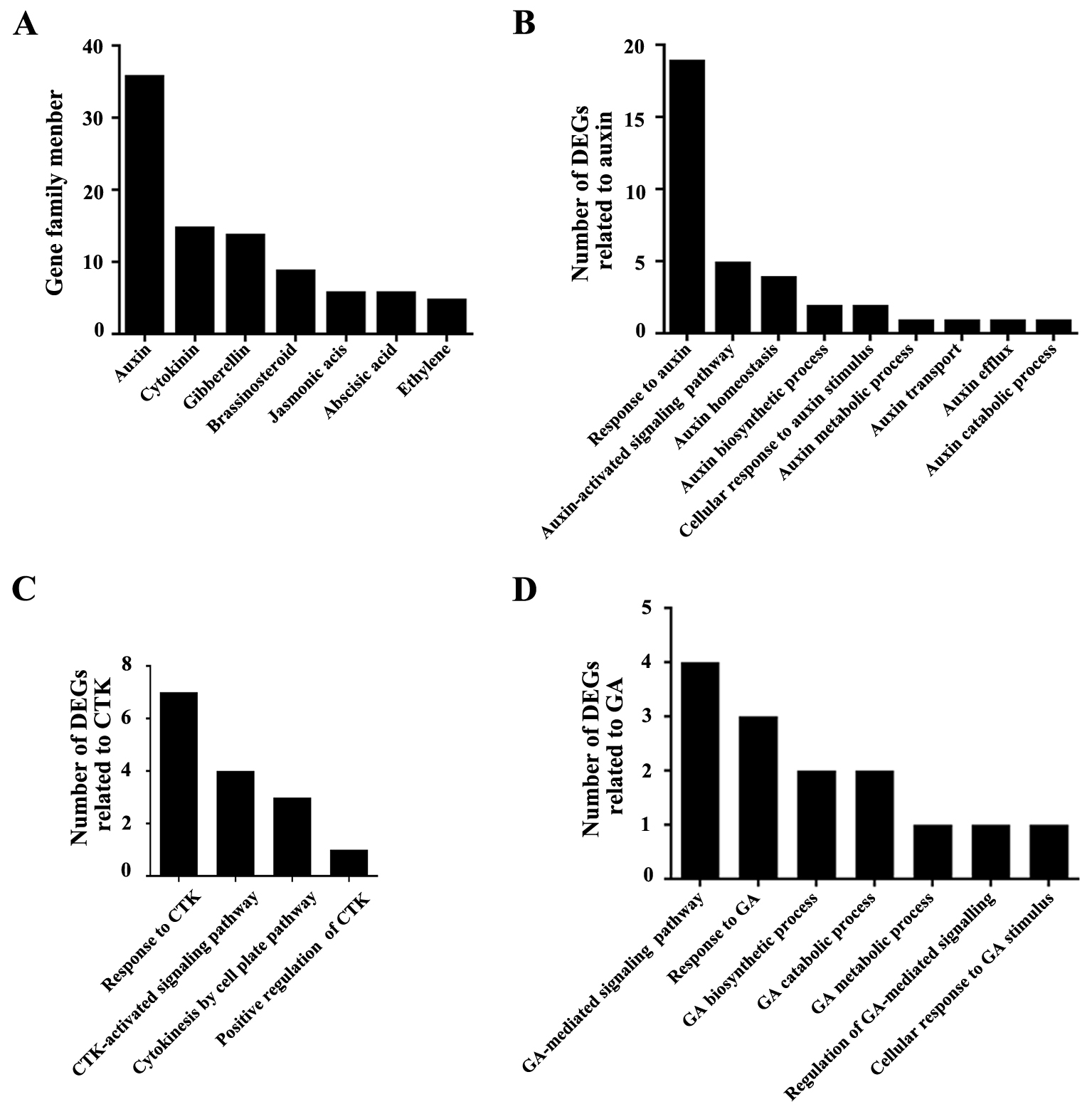


**Supplementary Figure 2. DEGs enriched in plant hormones.** A, the numbers of DEGs enriched in different hormone response pathways. B, DEGs enriched in auxin. C, DEGs enriched in cytokinin. C, DEGs enriched in gibberellin.


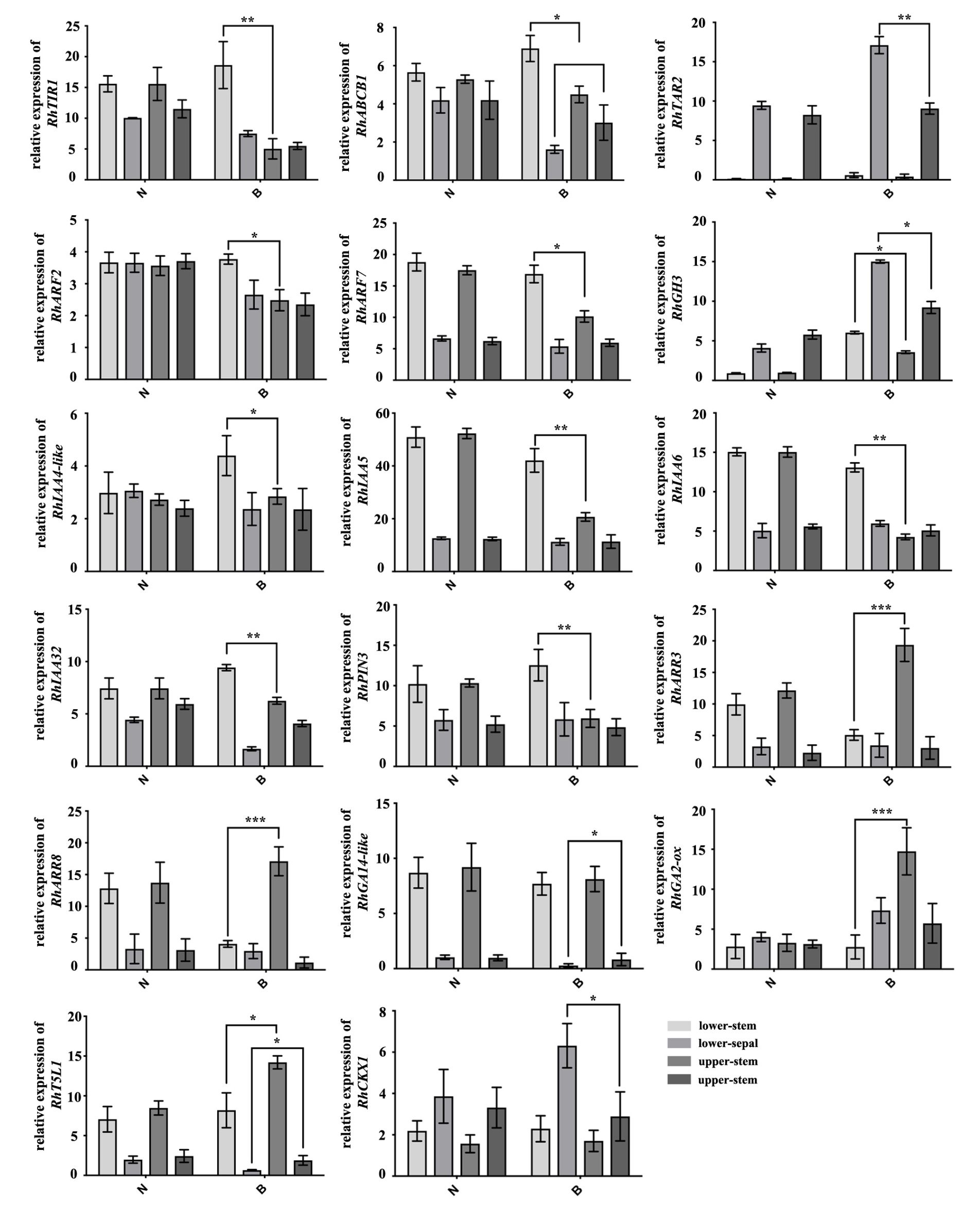


**Supplementary Figure 3. qRT-PCR of phytohormone-related genes in different tissues.**

Genes include those encoding auxin receptors (*RhTIR1*), auxin-responsive factors (*RhIAA4-like*, *RhIAA5*, *RhIAA6*, *RhIAA32*), auxin transporters (*RhPIN3*, *RhABCB1*) and auxin-regulated factors (*RhARF2*, *RhARF7*), cytokinin-related genes (*RhARR3,* *RhARR8, RhT5L1, RhCKX1*), gibberellin-related genes (*RhGA14-like, RhGA2-ox*). Error bars represent SD. Three biological replicates were performed. Asterisks denote statistically signiﬁcant differences determined by using Student’s *t*-test (**P* < 0.05, ***P* < 0.01, ****P* < 0.001).
